# Nursing students experienced academic emotions during education - a longitudinal descriptive study from a nursing bachelor’s program in Sweden

**DOI:** 10.1186/s12912-024-01729-y

**Published:** 2024-01-18

**Authors:** Susanne Lundell Rudberg, Taina Sormunen, Max Scheja, Hanna Lachmann, Margareta Westerbotn

**Affiliations:** 1 Department of Learning, Informatics, Management and Ethics, Karolinska University, Stockholm, 171 77 Sweden; 2 Department of Health Promoting Science, Sophiahemmet University, P. O. Box 5605, Stockholm, 114 86 Sweden; 3 Department of Nursing Science, Sophiahemmet University, P. O. Box 5605, Stockholm, 114 86 Sweden; 4 Department of Education of Stockholm University, Stockholm, 106 91 Sweden; 5 Department of Clinical Science and Education, Södersjukhuset, Karolinska Institutet, Stockholm, 118 83 Sweden

**Keywords:** Academic emotions, Contextual activity sampling system, Ongoing learning activities, Students, nursing, Longitudinal study

## Abstract

**Aim:**

To explore nursing students’ academic emotions during ongoing learning activities focusing on perceived challenge and competence.

**Background:**

Emotions plays an important part in learning. Positive emotions can be beneficial while negative emotions can be detrimental to educational outcomes. Optimal experiences are situations when learners simultaneously experience sufficient challenge and competence. Since various learning activities are performed in different learning environments during the nursing program, it is of interest to investigate students’ ongoing emotions in the occurring contexts.

**Design:**

A longitudinal descriptive study.

**Methods:**

By using the Contextual Activity Sampling System, data was collected every third week on a three-year nursing program. From August 2015 to January 2020, a total of 2, 947 questionnaires were answered by 158 students. Experiences of positive and negative academic emotions were calculated for the entire program. Optimal experience was calculated for courses where high discrepancy between positive and negative experiences were identified.

**Results:**

Students self-reported academic emotions varied over time and in relation to learning activities. High ratings of negative emotions were reported during clinical practice in all semesters except the final. Students’ positive academic emotions and optimal experience in clinical practice increased after having deepened their academic knowledge.

**Conclusion:**

Nursing students had an increased positive experience when they themselves practice a learning activity and it appeared that they benefit from academic preparation prior to entering internship. Nursing students need an academic competence to develop their skills during training in the clinical reality. Increased collaboration between academia and clinic would be beneficial for students’ clinical development.

## Introduction

Emotions play an important role in learning [1, 2]. Emotions can both promote and impede how individuals interact with the world around them [3]. Enjoyment of achievement in academic activities can enhance academic performance while the experience of boredom and anger can be detrimental to educational outcomes [2]. Furthermore, the presence of positive psychosocial learning environments are a significant in predicting that students complete their studies in higher education [4]. During their studies, students commonly experience so-called ‘academic emotions’, e.g., enjoyment, hope, pride, relief, anger, anxiety, shame, hopelessness, and boredom [5]. Such academic emotions can have a significant impact on the students’ memory, problem-solving abilities, and motivation to learn [2, 6]. During the education nursing students are required to develop nursing proficiency to work successfully in a clinical context after graduating. Bedside care performed by competent professional nurses are associated with better outcome for patients [7]. Enrolment requirements for Nurse education and to become a Registered Nurses [RN], vary globally with the governance of nursing education controlled and regulated by national frameworks [8]. Furthermore, nursing students are educated and trained in various context, often academically educated at universities and practically trained in clinics [9]. Previous research has often investigated students’ experiences after they have completed their education. A few studies have investigated the level of self-assessed competence of nursing students and newly graduated nurses [10–12]. However, less is known about students’ experienced emotions during theoretical education and clinical training throughout the nursing educational program. Since emotions can plays an important role in the learning process this study aimed to explore nursing students’ academic emotions during ongoing learning activities focusing on perceived challenge and competence.

## Methods

### Design

A longitudinal descriptive study.

### Settings and sample

This study was carried out within a nursing program at a university in Sweden. The program led to a professional degree as a RN, as well as a bachelor’s degree. This nursing program was in line with the national guidelines involving three-years of full-time study (equivalent to 180 credits, according to the European Credit Transfer and Accumulation System, ECTS), of which clinical practice accounted for 60 ECTS. The main subject, nursing science, corresponded to 109 ECTS credits and medical science 71 ECTS credits. The first year of the program consisted of theoretical studies, the second year included theoretical studies and clinical training. The third and final year mainly consisted of clinical training, except for completing a bachelor’s thesis in the fifth semester and theoretical education in leadership in semester 6, Table 1.

**Table 1:** Overview of courses at the university’s nursing program

Week	1	2	3	4	5	6	7	8	9	10	11	12	13	14	15	16	17	18	19	20
Semester 1	Nursing science					Medical science								Nursing science					Medical science	
Semester 2	Nursing science																			
Semester 3	Nursing science			Clinical training elderly care					Clinical training medical care					Clinical training surgical care					Nursing science	
Semester 4	CPE*	Clinical training psychiatric care					Nursing science						Research methodology				Nursing science			
Semester 5	CPE*		Clinical training primary health care				Clinical training palliative care				Writing bachelor’s thesis									
Semester 6	Leadership and pedagogics							Clinical training Interprofessional education		Clinical training advanced surgical care				Clinical training advanced medical care				Clinical training OR		Leadership

*Clinical Preparatory Education

Inclusion criteria were (a) admitted to the program, (b) having started the education. Exclusion criteria were (a) not signing informed consent, (b) drop out of the education. Included students who took a study break and started the education again during data collection were offered to resume participation in the study.

### Data collection via CASS questionnaires

The six-semester nursing program started twice a year. All students who started the program from August 2015 to February 2017 were invited to participate in the study. Out of 450 eligible students 158 signed an informed consent to participate in the study. Data was collected by using Contextual Activity Sampling System, CASS [13–15], that has been tested and validated for a Swedish context [16]. Participating students received a CASS-questionnaire via the universities learning platform every third week during the program. Each student received seven questionnaires per semester, a total of 42 questionnaires. One questionnaire took approximately 3 to 4 min to complete and included 12 questions. A total of 2,947 questionnaires were completed, Table 2.

**Table 2:** Presentation of number of answered questionnaires

Semester	Start of the nursing program				Total of questionnaires	Participating students
	Aug-15	Jan-16	Aug-16	Jan-17		
1	262	144	142	200	**748**	158
2	190	91	118	184	**583**	93
3	120	75	106	142	**443**	68
4	97	73	102	143	**415**	60
5	89	74	98	140	**401**	60
6	78	64	90	125	**357**	54
**Total of questionnaires**	**836**	**521**	**656**	**934**	**2,947**	-

The CASS questionnaires contained the same questions at each measuring point. The four opening questions dealt with which course the student attended, which learning activity was perceived as most important at the time and whether any collaboration with others was ongoing. These were multiple choice question with possibility of free text answers. In the following eight questions students self-assessed and reported their experienced emotions. Six questions covered a self-assessment of experienced positive emotions (determination, enthusiasm, interest) and negative emotions (irritation, nervousness, anxiety) originated from the PANAS scale [17, 18]. In the last two questions students were requested to rate perceived challenge and competence related to their current learning activity; combined responses to these two questions indicated the level of optimal experience, so-called ‘flow’ [19–21]. A seven-point Likert scale, ranging from 1 = strongly disagree to 7 = strongly agree, was used for rating experienced emotions, perceived competence and challenge [22].

According to the four-channel model an optimal experience involves so-called ‘flow’, situations when learners experience a task as challenging yet have adequate competence to manage it [19, 21]. The state of ‘flow’ is experienced when individuals are absorbed in an activity, feeling great pleasure while competing the task and losing a sense of time [20]. A high level of challenge in combination with low level of competence results in anxiety, while a low level of challenge and high level of competence results in boredom. Further a low level of challenge and low level of competence results in apathy [21].

### Analysis

The Statistical Package for Social Sciences, SPSS 27.0, and Microsoft Excel were used for the statistical data analysis. To reduce the effects of variances related to individual answering tendencies, Z-scores were standardized for each student and for each question by setting the mean to 0 and the SD to 1 [23]. Experiences of positive and negative academic emotions were calculated for the entire program. For courses with a marked difference between positive and negative emotions students optimal experience were calculated. Z-scores for competence and challenge were used to determine the individual position in the four-channel model: apathy (both challenge and competence below average); boredom (challenge below and competence above average); anxiety (challenge above and competence below average) and optimal experience “flow” (both challenge and competence above average).

## Results

### Demographics

At baseline, the sample (*n* = 158) was made up of 87.3% women and 12.7% men with a mean age of 27.8 ± 8.5, range 19–55. The nursing program was the primary choice for 90.5%, and 57% had family members working in health care. A total of 42.4% had previously attended higher education, and 30% had previous experience of completing a university degree in another subject area.

### Academic emotions

**Fig. 1:**
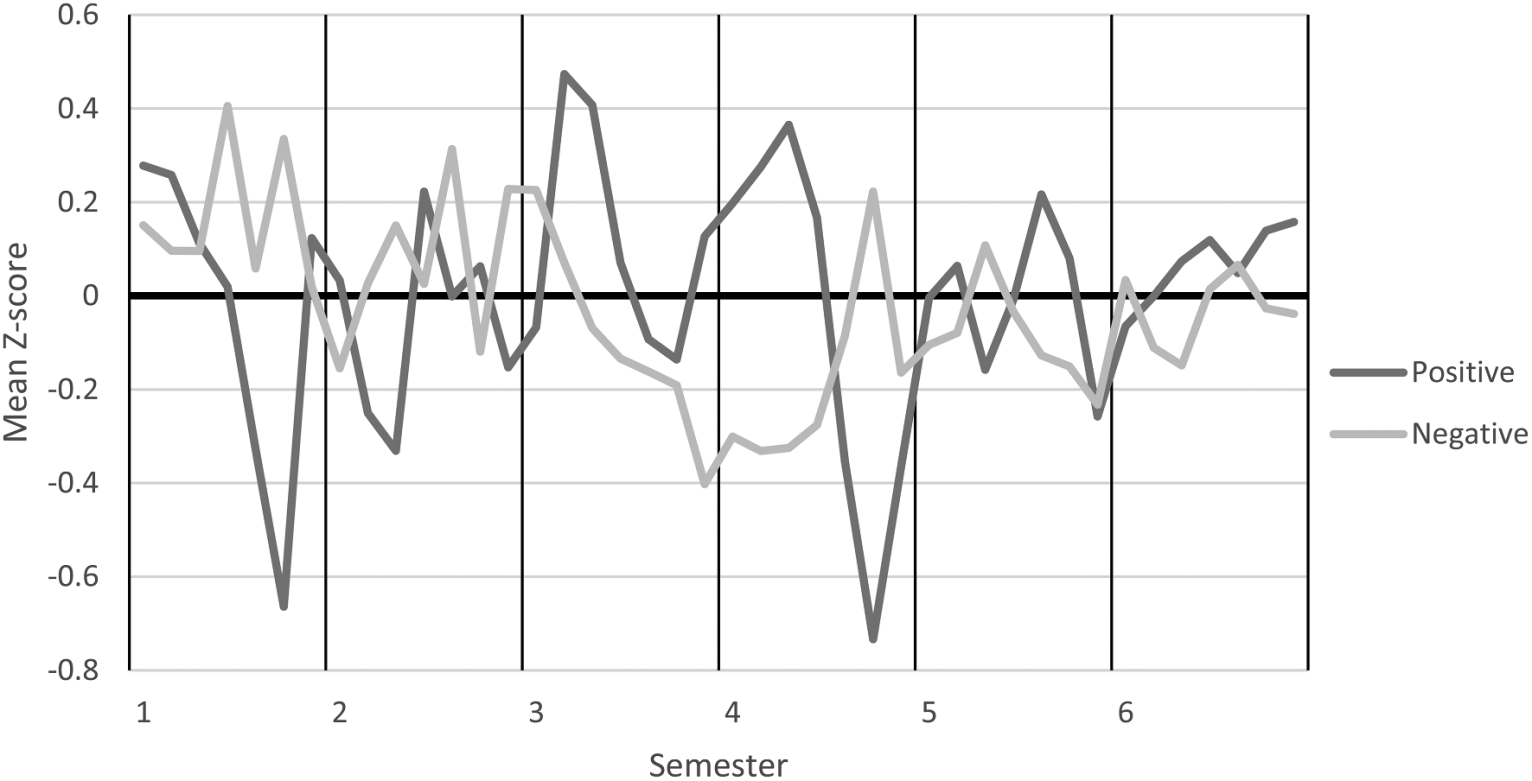
Mean Z-scores of changes in positive and negative academic emotions during education

Results of high positive academic emotions combined with low negative emotions were reported when students first entered clinical practice in the third semester, upon completion of clinical practice in the fourth semester and while writing their bachelor thesis in the fifth semester. Ratings of low positive emotions and high negative emotions were reported during theoretical courses in medical science and in research methodology. The discrepancy between positive and negative academic emotions was reduced in the final year, revealing more positive emotions compared to negative emotions at the time of graduation, Fig. 1.

### Experiences of competence and challenge

**Fig. 2:**
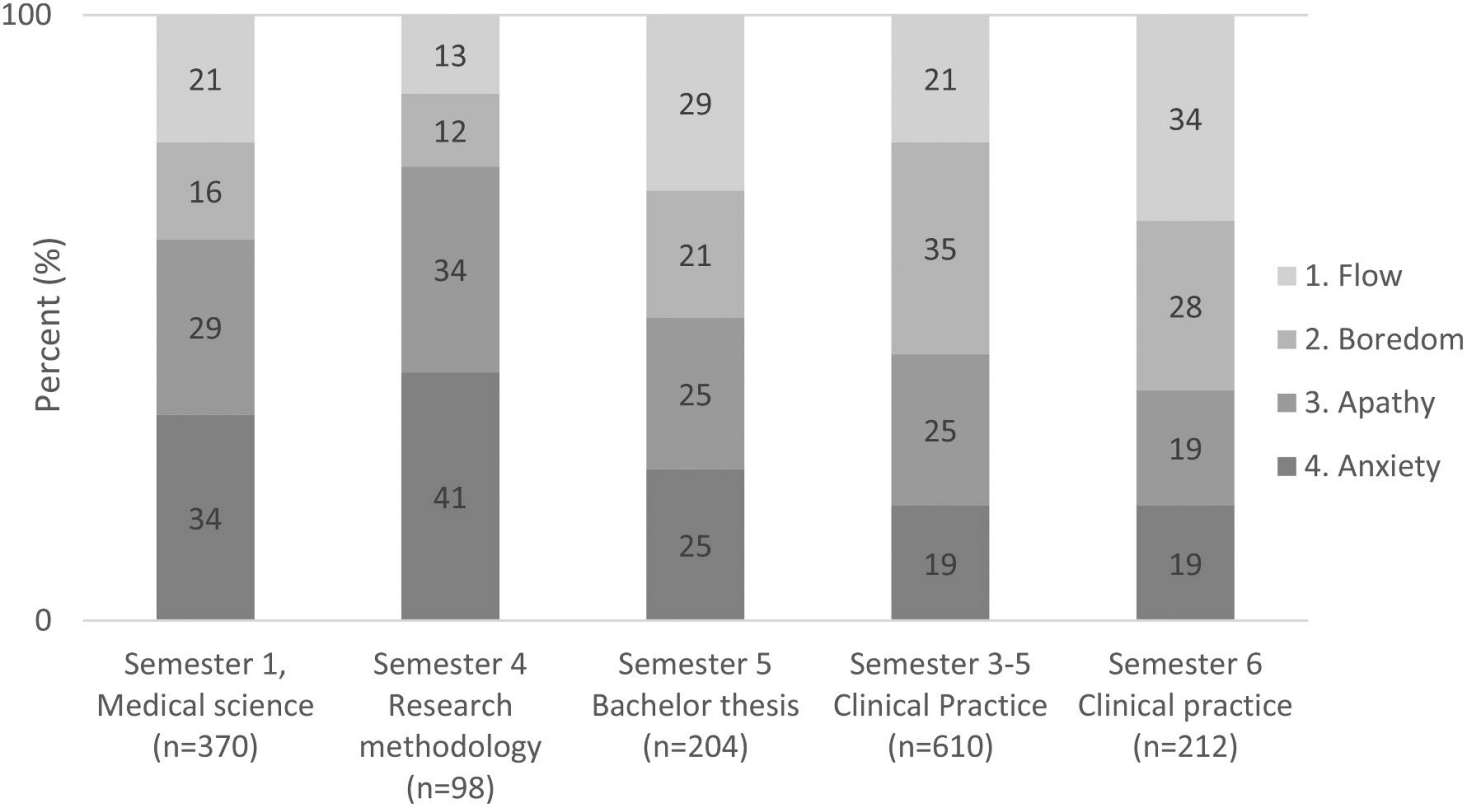
Experience of competence and challenge over time according to the four-channel model

High ratings of positive academic emotions together with low negative emotions were compliant with reported perceived challenge and competence. Analysis regarding competence and challenges were performed on the courses where the dichotomy between positive and negative emotions was prominent. Students’ experiences of competence and challenge decreased in semester one during the course in medical science and in semester four during the course in research methodology. The analysis concerning the four-channel model revealed a low percentage of ‘flow’ in these courses. During the process of writing their bachelor’s thesis, the range of ‘flow’ increased, with a reduction of boredom, apathy, and anxiety. Furthermore, a high level of boredom was reported during clinical practice in semesters three to five. The percentage of ‘flow’ was highest when writing bachelor’s thesis and during clinical practice in the final semester of the program, Fig. 2.

## Discussion

The aim of this study was to explore nursing students’ academic emotions during ongoing learning activities focusing on perceived challenge and competence. It has previously been found that applicants to nurse education are eager to learn and are full of enthusiasm [24]. Our results disclosed low levels of ‘flow’ together with high levels of anxiety and apathy during the medical research course, which aims at preparing students for writing up their bachelor´s theses. Henttonen et al., [25] have investigated nursing students’ experiences of the thesis writing process and underscore that students’ have a wide range of expectations and emotions prior to writing their bachelors thesis. Furthermore, according to our findings the state of ‘flow’ is largely achieved during the actual thesis writing, indicating that students subsequently develop their skills in the academic field when performing the task themselves.

There is a considerable diversity in the level and standard of nurse education both nationally and internationally [26]. Nurse education in the Nordic countries is required to follow the directive requirement of the European Union [EU]. Clinical practice must therefore cover a minimum of 2 300 h [90 ECTS] [27]. It is stated in article 31 [28] that: ‘Clinical practice is that part of nurse training in which trainee nurses learn, as part of a team and in direct contact with a healthy or sick individual and/or community, to organize, dispense and evaluate the required comprehensive nursing care’. These requirements indicate that half of the nurse education should be performed in clinical settings, therefore it is necessary that students experience a purposeful learning environment in the clinic. Today’s nursing students need education and training adapted to the clinical reality [9]. The high percentage of students experiencing boredom during initial clinical practice may indicate that students expected learning outcomes in clinical settings may not be fulfilled. A plausible explanation to experienced boredom is that the students considered themselves as having too high levels of competence and therefore did not find the clinical situations challenging enough. It could also be explained by the fact that they themselves were not able, or did not receive guidance, in identifying and reflecting on situations experienced. Another explanation could be that the students do not actually get the opportunity to perform nursing chores. It is conceivable that students may discover that clinical staff member do not always work according to guidelines and theories taught and learnt during the university studies. Moreover, factors relating to the clinical work environment have generated a nursing staff turnover that presents a serious challenge to health care [29]. The shortage of RNs may entail that students miss out on opportunities to participate in the clinical work, with limited prospects for guidance, supervision, and feedback from clinical staff. This problem can to some extent be alleviated by the universities preparing students for clinical practice through practice with patient simulators, digital training tools and exercises in pedagogical methods for reflection. Different types of simulation technology have been found effective in preparing nursing students for clinical work [30]. Nevertheless, simulation cannot replace clinical practice, however it might increase students’ preparedness for the clinical reality. Furthermore, generation Z (born 1996–2015) nursing students may be more grounded in technology driven, prefer to work in their own pace and desire more feedback compared to previous cohorts [31]. This means additional requirements for an appropriate education and training to be able to ensure the supply of nurses in the future health care.

## Conclusion

Students self-reported academic emotions varied over time and were linked to learning activities during their education and training progression. Students experienced academic emotions shifted from having negative emotions during the thesis preparation period, to more positive emotions during the thesis writing process. Accordingly, it seems that nursing students have an increased positive experience when they themselves practice a learning activity. The results of the academic emotions ratings combined with calculated ‘flow’ during clinical practice indicates a discrepancy between theoretical education and clinical training. Our findings disclosed that students’ positive academic emotions and experiences of ‘flow’ in clinical practice increased after having deepened their academic knowledge. There is a need for universities to prepare students for the contemporary clinical reality since it appears that students may benefit from additional academic preparation prior to entering internship. An increased collaboration between the faculty staff and clinical supervisors would be beneficial to students’ experiences of learning in clinic.

### Strengths and limitations

This study offered longitudinal insights into students self-reported academic emotions during different learning activities throughout their education. It is important to be mindful that reasons for changes in experienced emotions can include non-educational factors. Since the researchers were employed at the university, they were familiar with the learning environment and had access to the university learning platform to facilitate the data collection. To avoid influence from teachers, data collection and participation in the study was not discussed in the context of education A possible limitation of this study is that data were collected at a single university in Sweden; however, the Swedish nurse education program is regulated by national guidelines, which suggests that these findings may be of relevance to programs of a similar kind. All data were self-reported estimates based on predetermined concepts for selected academic emotions. For that reason, the meaning of the various concepts can be interpreted individually, which could affect the result. In order to compare results from different distributions and to reduce the number of extreme estimates, the Z-score was used in the analyses. Further research to explore factors associated with students experienced academic emotions is needed to identify improvement possibilities regarding the study environment at both universities and in clinical placements.

## Data Availability

No datasets were generated or analysed during the current study.
